# Owner Awareness, Motivation and Ethical Considerations in the Choice of Brachycephalic Breeds: Evidence from an Italian Veterinary Teaching Hospital Survey

**DOI:** 10.3390/ani15152288

**Published:** 2025-08-05

**Authors:** Giovanna Martelli, Fabio Ostanello, Margherita Capitelli, Marco Pietra

**Affiliations:** 1 Department of Veterinary Medical Sciences, University of Bologna, 40064 Ozzano dell’Emilia, BO, Italy; giovanna.martelli@unibo.it (G.M.); fabio.ostanello@unibo.it (F.O.); 2 Independent Researcher, 40100 Bologna, BO, Italy; margherita.capitelli@libero.it

**Keywords:** extreme traits, dog welfare, veterinary counseling, brachycephalic breeds, owner demography, emotional decision-making, dog owner behavior, health awareness, university veterinary hospital

## Abstract

The recent surge in the popularity of brachycephalic dog breeds has raised concerns about their predisposition to serious health issues linked to breed-specific morphological traits. This study examined the demographic characteristics, motivations, and awareness of owners regarding welfare issues in four brachycephalic breeds (French Bulldogs, Bulldogs, Pugs, Boston Terriers). Methods: A total of 497 owners of brachycephalic dogs examined over six years at an Italian university veterinary hospital were considered; a subset of 75 owners completed a structured questionnaire. Based on responses to a key multiple-choice question about the main reason for breed choice, owners were classified into three groups: trend-driven (aesthetics/fashion), value-oriented (intelligence/behavior), and indeterminate. Results: Gender distribution did not differ significantly compared to the overall population, but brachycephalic owners were significantly younger (*p* < 0.001). Value-oriented owners were significantly more likely (*p* < 0.01) to consult a veterinarian before acquisition and showed better understanding of typical respiratory issues, which did not affect their purchasing decision. Trend-driven owners were more influenced by public figures (*p* < 0.05) and less engaged in preventive care. Conclusion: Our findings highlight the need for pre-acquisition veterinary counseling. Veterinarians can also assist breeders by promoting awareness of the ethical risks of selecting extreme traits in dogs.

## Simple Summary

Brachycephalic dog breeds are increasingly popular worldwide due to their distinctive appearance and calm temperament, often considered ideal for domestic life. However, these same features are associated with serious health problems, such as Brachycephalic Obstructive Airway Syndrome, which may compromise animal welfare. This study examined demographics, motivations, and awareness of owners of brachycephalic dogs (French Bulldogs, Bulldogs, Pugs, and Boston Terriers) examined at an Italian university veterinary teaching hospital. Based on their main reason for choosing the breed, owners were grouped as trend-driven (aesthetics/fashion), value-driven (intelligence/behavior), or indeterminate (i.e., not classifiable within the previous categories). While gender distribution did not differ significantly from the general population, brachycephalic dog owners were significantly younger. Value-driven owners were more likely to seek veterinary advice before acquisition and showed greater awareness of breed-related health issues. In contrast, trend-driven owners were more influenced by media and fashion and showed lower engagement in preventive actions. Almost all owners, regardless of group, sought veterinary care after acquiring the dog, suggesting increased concern once emotional bonds are formed. These findings highlight the importance of promoting evidence-based veterinary counseling before purchasing a dog. Veterinarians could also support breeders by raising awareness of the ethical implications of selecting extreme traits, ultimately helping to reduce the welfare burden linked to these breeds.

## 1. Introduction

In recent decades, brachycephalic breeds, especially those of small size, have exhibited a marked increase in popularity worldwide. Among them, the French Bulldog has experienced the most significant demographic expansion. In the United States, it has been the most popular dog breed registered with the American Kennel Club (AKC) [1] from 2022 to the present, overtaking the Labrador Retriever after a thirty-year dominance. A similar trend has been observed in the United Kingdom, where the breed rose from the fourteenth most registered in 2013 to third place in 2024, behind the Labrador Retriever and the Cocker Spaniel and overtaking the Golden Retriever and the Miniature Dachshund [2,3]. Despite the global popularity of brachycephalic dogs, attraction to these breeds is not a modern phenomenon. Onar et al. [4] reported the discovery of a brachycephalic dog skull buried in a necropolis in the ancient city of Tralleis, Turkey, dating back approximately 2000 years. This finding suggests that a preference for dogs with such distinctive facial features existed even at that time and that they were likely valued as close human companions.

These dogs are characterized by a distinctive craniofacial morphology, including a short muzzle, broad skull, and prominent forehead combined with temperamental traits generally perceived as well-suited to domestic life. Regarding aesthetics, Hecth et al. [5] and Paul et al. [6] highlighted that brachycephalic breeds possess neotenic facial features—such as a rounded head, prominent forehead, and large eyes—which evoke strong feelings of affection and protection in humans. This phenomenon is comparable to the innate human response to newborns, explaining the emotional and psychological appeal of these breeds often influencing purchasing decisions more than functional or health-related considerations.

However, as the popularity and demographic expansion of these breeds continue to rise, it is reasonable to expect a corresponding increase in the prevalence of breed-related health issues closely linked to the effects of artificial selection for extreme craniofacial traits [7,8]. These include respiratory diseases, ocular abnormalities and complications, including chronic hypoxia, and systemic hypertension. Among them, Brachycephalic Obstructive Airway Syndrome (BOAS) represents the most emblematic and widely studied condition, characterized by anatomical abnormalities such as stenotic nares, presence of aberrant nasal turbinates, macroglossia, elongation of the soft palate, laryngeal collapse, and tracheal hypoplasia, all of which compromise breathing and significantly reduce the affected dogs’ quality of life. While surgery is often considered the primary treatment for BOAS [9], studies indicate that the syndrome has broader and more complex implications [10,11]. In addition to respiratory difficulties, brachycephalic dogs may suffer from sleep disturbances, chronic hypoxia, systemic hypertension, and gastrointestinal disorders, including cardial atony with gastroesophageal reflux and distal esophagitis, hiatal hernia, and pyloric hyperplasia [12]. Another possible malformation in some of these breeds is brachicephalic ocular syndrome, which is characterized by a shallow orbit combined with euryblepharon, leading to issues such as ‘scleral show’, entropion, trichiasis, exotropia, lagophthalmos, reduced corneal sensation, and a compromised tear film [13], as well as the presence of a malformation of the vertebrae (hemivertebrae) [14]. These conditions further compromise their overall well-being. These findings highlight the necessity of viewing BOAS not merely as a surgical issue but as a systemic disorder requiring a comprehensive approach to canine health management. This approach should include genetic prevention, increased owner awareness, and selective breeding practices aimed at improving the overall health of these breeds [15].

In response to health harms caused by such extreme conformations, the European Commission presented on 7 December 2023 its proposal for a regulation on the welfare of dogs and cats and their traceability [16]. This proposal establishes, for the first time, EU-wide minimum standards for breeding establishments, pet shops, and shelters, covering housing, identification, traceability, veterinary oversight, and, importantly, breeding strategies that prevent phenotypes detrimental to health. However, as repeatedly emphasized by Packer et al. [17,18] and more recently reiterated by Cannas et al. [19], one of the most critical aspects of BOAS management is owners’ perception of the problem. In fact, these studies suggest that many owners tend to normalize their dogs’ respiratory symptoms—such as snoring, thermoregulation difficulties, and exercise intolerance—mistaking them for intrinsic breed characteristics rather than clinical signs of suffering. Some even minimize or rationalize these health issues to avoid cognitive dissonance between the emotional attachment to their pet and the awareness of its suffering. This misperception can delay veterinary intervention and perpetuate the selection of dogs with severely compromised conformations, fueling a vicious cycle in which aesthetics takes precedence over animal health and welfare.

Against this background, the present study aimed to investigate the demographic characteristics, motivations, and welfare awareness of Italian owners of brachycephalic dogs, using data collected at a university veterinary teaching hospital over the last six years. Owners were also categorized into different typologies based on their reasons for choosing the breed. The study was conducted on only four breeds, namely French Bulldog, Pug, English and American Bulldog, and Boston Terrier, and not on all brachycephalic breeds, as these four breads are more prominently associated with brachycephalic syndrome in the literature [20,21] and have shown a notable increase in popularity both globally and in Italy [22].

By identifying differences in owner profiles, knowledge, and behavior, this study seeks to inform public education strategies, support veterinary counseling, and contribute to ethical breeding practices aimed at reducing the demand for extreme morphologies.

## 2. Materials and Methods

### 2.1. Data Collection

This study included patients examined at the Veterinary Teaching Hospital of the Department of Veterinary Medical Sciences at the University of Bologna (Northern Italy), between January 2019 and December 2024.

The selection of brachycephalic dogs, namely French Bulldog, Bulldog (meaning both English and American Bulldog), Pugs, and Boston Terrier, was conducted by searching the hospital database for admissions (Group A, no. 497). Data on dogs of these brachycephalic breeds were included only if the breed had more than 10 individuals recorded in the period.

For each dog, its breed was recorded, and if multiple visits occurred during the examination period, only the first visit was included to avoid overestimation.

For each selected dog, the owner’s sex and age at the time of the visit and personal email were retrieved from the hospital database.

Cases where the owner’s reporting was incomplete (i.e., missing sex or age), as well as cases where the owner was a company rather than an individual, were excluded.

As a control group (Group B, no. 10397), data from all dogs observed during the same period were included, excluding the animals from Group A and considering only first-time visits.

To assess potential differences in the sex and age of the owners in Group A compared to those of owners of other canine breeds presented to the facility during the same period (Group B), the demographic records of each owner, stored in the Hospital Database, were utilized.

Owners of Group A, identified in the database, were sent a questionnaire via the email address they had provided, with authorization for the use of sensitive data from the hospital.

The questionnaire consisted of 1 multiple-choice and 12 binary questions with fixed answer options, created using an online form tool and designed to be completed anonymously, in compliance with European Union privacy regulations [23]. The questionnaire was specifically designed for this study and was neither pilot-tested nor derived from previously validated instruments. Submission was only possible once all questions had been completed. All the responses were anonymous, and it was not possible to identify the age, sex, or any other sensitive data of the respondents.

The owners were informed about the objectives of the study and that their responses would contribute to the research.

Completing the test required 2 to 5 min of the owner’s attention. A total of 497 questionnaires were sent; 75 owners submitted their responses (15.1%). The study was part of the regular clinical activity of the Veterinary Teaching Hospital of the University of Bologna and did not involve any handling or procedures on animals beyond those required during a routine initial clinical examination at the reference facility; accordingly, ethical approval for animal experimentation was not deemed necessary. However, prior to the study, all dog owners provided informed written consent by signing a dedicated authorization form (File S1), which explicitly permitted the scientific use of the collected data in full compliance with European privacy regulations [23]. Besides guaranteeing respondents’ anonymity, the procedure ensured that all information would be handled in accordance with applicable privacy laws.

### 2.2. Design of the Questionnaire

The questionnaire included the following questions (the possible answers are in parentheses):

1. What was the main reason for choosing a brachycephalic breed? (aesthetics/fashion; intelligence/behavior; other)
2. Given the current popularity of this breed, do you think your choice may have been influenced by public figures who own brachycephalic dogs? (No; Yes)
3. Are you aware of the genetic modifications affecting this breed? (No; Yes)
4. Did you consult a veterinarian before purchasing your dog to learn about proper breed management? (No; Yes)
5. Did you consult a veterinarian after purchasing your dog to learn about proper breed management? (No; Yes)
6. Are you aware that this breed may have nasal stenosis (narrow nostrils that restrict airflow)? (No; Yes)
7. Are you aware that these breeds may have an elongated soft palate, which can cause breathing difficulties? (No; Yes)
8. Are you aware that these breeds may suffer from tracheal hypoplasia (smaller trachea), leading to breathing difficulties? (No; Yes)
9. Have you ever had specific diagnostic tests performed to assess breathing problems (e.g., X-ray, endoscopy, CT scan)? (No; Yes)
10. Are you aware that surgery may be an option to address these breathing issues? (No; Yes)
11. Have you already opted for surgical procedures? (No; Yes)
12. Would prior knowledge of these respiratory symptoms have influenced your decision to choose this breed? (No; Yes)
13. Would knowledge of the costs associated with these procedures have influenced your purchasing decision? (No; Yes)

Question 1 was designed to provide the initial categorization of brachycephalic dog owners by dividing them into three groups. The three response categories were developed a priori based on common motivational themes reported in the literature [24,25]. The first group comprised individuals whose choice of a brachycephalic breed was primarily influenced by social factors or by an attraction to the dog’s distinctive appearance (response: aesthetics/fashion). These respondents were classified as trend-driven owners.

The second group consisted of owners whose motivations went beyond the physical appearance of the dog (response: intelligence/behavior), indicating a focus on qualities such as temperament and overall compatibility with their lifestyle. These individuals were categorized as value-oriented owners, as their choices appeared to be driven by intrinsic characteristics they expected or desired in the animal, prioritizing stable and enduring traits over external appeal.

The category ‘Other’ was intentionally not further investigated due to the wide range of possible motivations it could encompass. However, in line with Holland et al. [24], the predominant reason was likely related to contextual factors, namely previous personal experiences and the influence of friends and family. Therefore, this third group included respondents whose stated reasons did not align clearly with either of the previous categories and were designated as indeterminate owners.

This composite classification was guided by logical consistency and aimed at enhancing the interpretability of the results. Similar approaches are commonly used in studies involving behavioral and attitudinal data [26].

The remaining 12 questions were developed to further explore the factors influencing the owners’ choice (Q2, Q12 and Q13), their level of awareness regarding health issues both prior to and following acquisition (Q3, Q6, Q7 and Q8), and their propensity to seek veterinary consultation both before and after the acquisition, as well as to pursue veterinary procedures (Q4, Q5, Q9, Q10, and Q11).

### 2.3. Statistical Analyses

Preliminary analyses were conducted to assess potential differences in gender distribution (male and female) and age among dog owners of Group A and Group B. Subsequently, questionnaire responses were analyzed, using the answers to Question 1 (what was the main reason for choosing a brachycephalic breed?) to categorize owners into three groups based on the criteria through which they had chosen a brachycephalic breed.

For the qualitative variable (gender of the owner), the chi-square test was used, while for quantitative variables (age of the owner), after assessing the normality of the sample distribution, either the Mann–Whitney U test or the Kruskal–Wallis H test was applied. Non-parametric tests were employed due to the limited sample size and the non-normal distribution of the data, as determined by the Kolmogorov–Smirnov test, which indicated a statistically significant deviation from a Gaussian distribution.

Statistical analyses were performed using the software SPSS 28.0 (IBM SPSS Statistics, New York, NY, USA). Significance was set at *p* < 0.05.

## 3. Results

### 3.1. Study Population

Within the cohort of the 497 owners identified, the distribution of brachycephalic dog breeds was as follows: 292 French Bulldog (58.8%), 101 Bulldogs (20.3%), 85 Pugs (17.1%), and 19 Boston Terriers (3.8%).

Among the owners of brachycephalic dogs, there were 191 males (38.4%) and 306 females (61.6%), with a median age of 49 years (range: 22–82 years) for males and 47 years (range: 21–91) for females (Table 1).

Among the other owners of these breeds (10,397), there were 4257 males (40.9%) and 6140 females (59.1%). The males had a median age of 57 years (range: 21–94 years), while the females had a median age of 53 years (range: 21–101) (Table 1).

**Figure 1:**
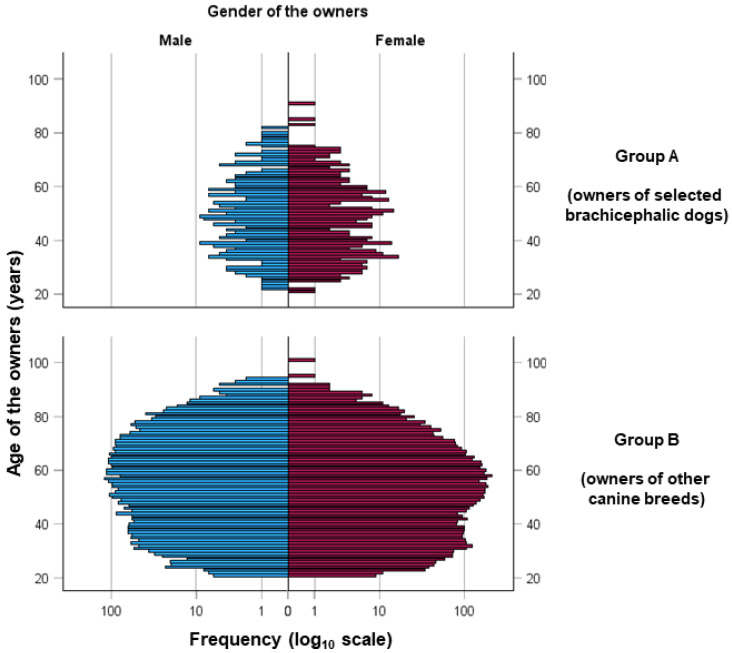
Demographic chart that illustrates the distribution of dog owners, categorized by the type of dog they own (selected brachycephalic breeds and other canine breeds) and segmented by the gender of the owners.

The two groups of owners did not differ significantly in terms of gender distribution (*p* = 0.265), whereas the owners of brachycephalic dogs were significantly younger (*p* < 0.001), as shown in Figure 1.

Among the owners of selected brachycephalic dogs, the gender distribution was relatively consistent for Boston Terriers, French Bulldogs, and Pugs, with females representing 63.2% (no. 12), 66.4% (no. 194), and 65.9% (no. 56) of owners, respectively. In contrast, the proportion of female owners was significantly lower (*p* < 0.001) among Bulldog owners (no. 44; 43.6%). Additionally, Bulldog owners were found to be significantly younger (*p* < 0.001) compared to owners of other selected brachycephalic breeds (median age: 46 and 48 years, respectively), as shown in Figure S1.

### 3.2. Questionnaire Analysis

A total of 75 responses to the questionnaire were received, corresponding to a response rate of 15.1%. Regarding Question 1 (what was the main reason for choosing a brachycephalic breed?), among all 75 respondents, 28.0% (no. 21) cited aesthetics/fashion, 41.3% (no. 31) intelligence/behavior, and 30.7% (no. 23) other reasons.

Following the methodological approach whereby owners were categorized into three groups based on their answers to Question 1, namely aesthetics/fashion (trend-driven owners), intelligence/behavior (value-driven owners), and other reasons, the corresponding distribution of respondents is reported in Table 2 and Table 3.

Regarding Question 2 (given the current popularity of this breed, do you think your choice may have been influenced by public figures who own brachycephalic dogs?), only trend-driven owners reported that their decision to acquire a brachycephalic dog was significantly influenced by the fact that public figures own or promote such breeds; this difference is statistically significant when compared to the other two owner categories (*p* = 0.018).

With regard to the level of knowledge concerning the genetic alterations characterizing various brachycephalic dog breeds (Question 3), no statistically significant differences were identified among the three owner groups (*p* = 0.319), with relatively high levels of awareness reported across the respondents (range: 81.0–95.7%).

The frequency of seeking veterinary advice prior to acquiring the dog, specifically to gain insight into the appropriate management of the breed, was significantly higher (*p* < 0.001) among owners who had chosen the breed based on intelligence-/behavior-related motivations (value-driven owners) compared to the other categories (Question 4). In contrast, no statistically significant differences were observed among the three groups regarding the use of veterinary consultations after the acquisition of the dog (Question 5; *p* = 0.350). Nonetheless, although the differences were not statistically significant, value-driven owners or those who made their choice for other reasons (indeterminate owners) exhibited a numerically greater propensity to consult a veterinarian (30 out of 31, 96.8%, and 21 out of 24, 91.3%, respectively) compared to trend-driven owners (18 out of 21, 85.7%).

As for knowledge of specific respiratory-related anatomical abnormalities, namely, stenotic nares (Question 6, Table 3), elongated soft palate (Question 7), and tracheal hypoplasia (Question 8), no statistically significant differences were found among the three owner categories (*p* > 0.05). However, although not significant, a higher degree of awareness of these conditions was generally observed among value-driven owners. Although the recourse to targeted clinical evaluations aimed at addressing respiratory issues (Question 9) did not differ significantly among the three groups (*p* = 0.144,), it is noteworthy that trend-driven owners reported a markedly lower frequency of such evaluations (8 out of 21, 38.1%) compared to value-driven and indeterminate owners (19 out of 31, 61.3% and 15 out of 23, 65.2%, respectively). A similar trend emerged regarding awareness of the possibility of surgical intervention for respiratory disorders (Question 10). Although the differences were not statistically significant (*p* = 0.190), trend-driven owners showed the lowest level of knowledge.

Regarding the possible use of surgical procedures (Question 11), no significant differences (*p* = 0.705) were found among the three groups of owners.

With respect to whether prior awareness of potential respiratory symptoms might have influenced the decision to acquire a brachycephalic dog (Question 12), a highly significant difference was observed among the three groups (*p* < 0.001): value-driven owners showed a very low likelihood of expressing regret over their purchasing decision even in light of their good knowledge of the typical health issues associated with brachycephalic breeds.

Finally, a tendency approaching statistical significance (*p* = 0.08) was observed suggesting that value-driven owners might be less inclined to consider prior knowledge of the impact of breed-related health care costs as a factor capable of influencing their breed choice (Question 13).

## 4. Discussion

### 4.1. Demographics

The response rate observed in our research, i.e., 15.1%, is lower than that reported in a similar study conducted in Denmark in 2015, which surveyed four small dog breeds (both with and without brachycephalic syndrome) and achieved a 29.4% response rate from 3000 owners [25]. However, the present findings align with those of Åsbjer et al. [27], who reported a 15.7% response rate among owners in a large-scale study on brachycephalic dogs’ health perceptions. Higher rates were observed among breeders (24.8%) and judges (41.7%), although the latter group was less represented numerically. Despite the variability among the respondent categories, this concordance with owners’ response rate observed in the aforementioned study supports the reliability of our data and confirms that response rates in our range are consistent with comparable recent studies.

Regarding the characteristics of some canine brachycephalic breed owners compared to those of the broader dog population admitted to the Veterinary Teaching Hospital of the University of Bologna during the same period, a similar proportion of female/male owners was observed, whereas the owners of brachycephalic dogs were significantly younger (*p* < 0.001). Existing studies suggest that younger individuals, particularly those under 29 years of age, are more inclined to select purebred dogs [28]. Consistent with the present findings, recent studies by Bognár and Kubinyi [29] and Packer et al. [30] have shown that brachycephalic dog owners are generally younger compared to owners of non-brachycephalic breeds. Moreover, given the well-documented impact of mass media on shaping dog breed demographics [31], younger individuals are especially susceptible to the influence of modern communication channels, including social media platforms, where brachycephalic breeds are frequently promoted by influencers and celebrities [32]. This heightened visibility and perceived social desirability may further contribute to the appeal of these breeds among younger generations.

Although women outnumbered men among brachycephalic dog owners in our sample, this gender difference was not statistically significant. This contrasts with findings by Bognár and Kubinyi [29], who reported a higher female preference for small brachycephalic breeds, likely attributable to the greater appeal that the infantile features typical of these dogs exert on a female audience [33]. The absence of significance in our case may therefore reflect the size and specific composition of our sample rather than a true conceptual divergence. Despite the absence of overall sex-based differences among brachycephalic dog owners, the relatively higher prevalence of male owners within the Bulldog subgroup can plausibly be related to the larger body size of this breed compared to the others considered in the study. This interpretation is supported by findings from a retrospective analysis of 955 dog adoptions in the Czech Republic [34], which revealed a significant gender-based difference in size preferences: while women adopted more small dogs, men were significantly more likely to adopt large ones, suggesting that breed size plays a relevant role in owner demographics. This aligns with qualitative research showing that individuals use breed and size selection as symbolic tools to express and reinforce their gender identity [35].

It should be noted that the recorded owner at the time of a dog’s admission may not always correspond to the person who chose the dog. For example, in some cases, the dog may be registered under a spouse’s insurance policy (a practice that has only recently gained popularity in Italy), or the actual owner may be too young to be listed. Nevertheless, this potential bias is unlikely to significantly affect the study, as it impacts both brachycephalic dogs and the control group equally.

### 4.2. Owners’ Motivation

Only a small but significant number of owners, all classified within the trend-driven group, acknowledged that public figures had influenced their choice of dog breed. This finding appears to be in apparent contradiction with the motivations they had previously declared, which were primarily based on aesthetics and/or fashion. Given that conforming to fashion trends or following the advice of public figures, particularly controversial ones such as certain influencers, may be perceived as superficial or unreflective behavior, it is plausible that the low number of acknowledgments can be attributed to the well-documented phenomenon of “social desirability bias” (SDB) [36]. According to SDB, individuals tend to modify their responses to align with what they perceive as socially acceptable norms or expectations rather than disclosing their genuine beliefs. This bias has been observed among pet owners such as Bir et al. [37], who found that respondents tended to downplay the importance of appearance and breed when reporting their own preferences but believed that “other people” (i.e., the average dog owner) considered these traits more important. This gap reflects a classic SDB pattern, where individuals try to present themselves in a more ethical or rational light. In our study, this may have also affected responses to health-related questions. Since brachycephalic disorders are increasingly perceived as ethically sensitive, some owners, regardless of their motivation group, may have overstated their awareness to align with what is considered appropriate or expected [17].

Moreover, clinical observations collected during this study revealed that most owners were not actually aware of the potential health issues affecting these breeds, including BOAS, nor of the associated veterinary costs. Respiratory noises were often misinterpreted as normal “snoring” rather than recognized as pathological signs. This contrasts with self-reported data, where most respondents claimed to be informed. The inconsistency highlights a gap between perceived and actual understanding, consistent with Packer et al. [17] and Cannas et al. [19], who found that many owners of dogs clinically affected by BOAS denied the presence of breathing problems despite evident symptoms. Such misperceptions may delay treatment and perpetuate the breeding of affected animals.

However, recent evidence suggests that some re-evaluation of public attitudes toward canine morphology may be underway. A recent UK-based study employing visual preference tests found that the general public tends to favor less extreme conformations over the exaggerated traits typical of brachycephalic breeds. Although these preferences are not yet fully mirrored in ownership trends, they signal a potentially important cultural transition with implications for future breeding and public awareness strategies [38].

### 4.3. Veterinary Engagement

In our study, a statistically significant difference emerged in favor of value-oriented owners, who more frequently reported having consulted a veterinarian prior to acquiring their brachycephalic dog. This result indicates that they represent a more mature and informed category of dog owners, characterized by a greater awareness of breed-specific welfare implications and a more proactive approach to responsible ownership. However, these findings appear to contrast with the results of Mead et al. [39], who, in a large-scale study of current and prospective dog owners in the UK, found that veterinarians were not among the most commonly consulted sources of information in the pre-acquisition phase, with websites, family and friends, and online forums being the predominant sources instead. The discrepancy observed between our findings and those of the aforementioned study may, at least in part, reflect contextual differences or characteristics specific to the study samples. Nonetheless, our results suggest that when veterinary input is sought, it is associated with more reflective and welfare-conscious acquisition choices. This underscores the need to strengthen the role of veterinarians in pre-acquisition counseling, particularly with regard to breed-related health and management implications. However, structural and perceptual barriers, such as time constraints, limited staffing, and concerns about client reactions, may hinder the consistent delivery of such counseling, potentially leading to moral distress and limiting the profession’s impact. Addressing these challenges calls for coordinated institutional support and clearer professional guidance [40].

Contrary to what was reported to have occurred prior to acquisition, the absence of significant differences between owner groups regarding post-acquisition veterinary consultation, combined with the fact that nearly all owners reported having consulted a veterinarian after acquiring their dog, suggests a general concern for the animal’s health once it has become part of the household. Although, to the best of the authors’ knowledge, no peer-reviewed studies have specifically investigated the relationship between the initial purchase cost of a dog and the owner’s willingness to incur subsequent veterinary expenses, our findings indicate two key aspects. First, owners demonstrate greater attentiveness to the animal’s health once an emotional bond has been established. Second, there appears to be a level of commitment to caring for an animal often acquired at considerable financial cost. This interpretation is indirectly supported by the absence of significant differences among the three owner groups regarding the performance of specific diagnostic tests (e.g., X-ray, endoscopy, computed tomography scans), with a general predominance of respondents reporting that such procedures had been carried out on their dogs. Similarly, no group differences were observed in the proportion of owners who had their dogs undergo corrective surgical procedures aimed at resolving respiratory abnormalities. However, it was not possible to determine whether the decision to proceed with surgery was driven by the actual clinical severity of the condition or by other factors.

The significantly lower influence of prior knowledge of potential health problems on the breed choice of value-oriented owners aligns with the consistently higher level of overall awareness demonstrated by this group throughout the questionnaire. Previous studies by Sandøe et al. [25] and Packer et al. [18] have shown that brachycephalic dog owners are generally inclined to repeat their breed decision when acquiring a new pet. Similarly, the tendency of value-oriented owners to report that anticipated knowledge of veterinary costs would not have influenced their purchasing decision appears to reinforce this inclination. However, as observed by Sandøe et al. [25] for French bulldog owners, the predicted probability for acquisition of the same breed tends to persist only if the first dog did not undergo significant welfare issues. As the number of such problems increases, the trend reverses: the probability of selecting the same breed again drops considerably after the first issue and falls below 5% after the fourth. This observation suggests that, despite their generally higher levels of awareness, value-oriented owners may still underestimate the long-term clinical and financial implications associated with these breeds, potentially tempering their otherwise well-informed decision-making approach.

## 5. Conclusions

Understanding how owner motivation relates to health awareness is key to improving the welfare of brachycephalic dogs. This study identified three owner profiles, namely trend-driven, value-oriented, and indeterminate, with value-oriented owners demonstrating the highest levels of awareness and pre-acquisition veterinary engagement. In contrast, trend-driven owners appeared less informed, while a considerable number of respondents fell into an indeterminate category with motivations that were difficult to clearly define. This represents the main limitation of the study and reflects the inherent complexity of owner typification. More broadly, it highlights a common methodological challenge in questionnaire-based research: balancing the depth required to explore nuanced motivational drivers with the conciseness necessary to ensure adequate response rates. Future studies employing qualitative or mixed-method approaches may help to further disentangle this complexity and provide a more detailed understanding of owner motivations.

In this multifaceted context, veterinarians hold a central and strategic role. Beyond clinical care, they are uniquely positioned to guide prospective owners through pre-acquisition counseling and to collaborate with breeders in promoting health-conscious and ethically sustainable practices. Their independent, evidence-based input serves as a critical counterbalance to emotionally or commercially driven choices.

While forthcoming EU legislation introduces important regulatory standards for companion animal welfare, legal provisions alone are unlikely to trigger immediate cultural change. Long-term progress will depend on a broader shift in owner and breeder attitudes. This underscores the need for coordinated institutional support, clearer professional guidance, and widespread recognition of the pivotal role veterinarians play in promoting informed and ethically responsible ownership.

## Figures and Tables

**Table 1:** Demographic profiles of dog owners, categorized by the breeds or types of dogs they own.

Breeds or Types of Dogs That Are Owned	Descriptive Statistics	Gender of Owner		Total
		Male	Female	
French Bulldog	no. of owners (%)	98 (33.6)	194 (66.4)	292
	Median age (years)	46	47	47
	Range (min–max)	24–79	21–91	21–91
Bulldogs	no. of owners (%)	57 (56.4)	44 (43.6)	101
	Median age (years)	49	40	46
	Range (min–max)	22–82	26–74	22–82
Pugs	no. of owners (%)	29 (34.1)	56 (65.9)	85
	Median age (years)	51	48	50
	Range (min–max)	30–76	27–85	27–85
Boston Terriers	no. of owners (%)	7 (36.8)	12 (63.2)	19
	Median age (years)	52	56	56
	Range (min–max)	32–70	45–71	32–71
Selected brachycephalic dog breeds (Group A)	no. of owners (%)	191 (38.4)	306 (61.6)	497
	Median age (years)	49	47	48
	Range (min–max)	22–82	21–91	21–91
Other canine breeds (Group B)	no. of owners (%)	4257 (40.9)	6140 (59.1)	10,397
	Median age (years)	57	53	54
	Range (min–max)	21–94	21–101	21–101

**Table 2:** Cross-tabulation of owners based on their answer to Question 1 (reason for choosing the breed) and their responses to the questions regarding veterinary engagement and motivations (Q2–Q5). The percentages are shown in parentheses.

	Questions	Groups	Yes	No	*p*
2	Given the current popularity of this breed, do you think your choice may have been influenced by public figures who own brachycephalic dogs?	Aesthetics/Fashion	3 (14.3)	18 (85.7)	0.018
		Intelligence/Behavior	0 (0.0)	31 (100)	
		Other ^1^	0 (0.0)	23 (100)	
		Total	3 (4.0)	72 (96.0)	
3	Are you aware of the genetic modifications affecting this breed?	Aesthetics/Fashion	17 (81.0)	4 (19.0)	0.319
		Intelligence/Behavior	27 (87.1)	4 (12.9)	
		Other ^1^	22 (95.7)	1 (4.3)	
		Total	66 (88.0)	9 (12.0)	
4	Did you consult a veterinarian before purchasing your dog to learn about proper breed management?	Aesthetics/Fashion	5 (23.8)	16 (76.2)	<0.001
		Intelligence/Behavior	21 (67.7)	10 (32.3)	
		Other ^1^	1 (4.3)	22 (95.7)	
		Total	27 (36.0)	48 (64.0)	
5	Did you consult a veterinarian after purchasing your dog to learn about proper breed management?	Aesthetics/Fashion	18 (85.7)	3 (14.3)	0.350
		Intelligence/Behavior	30 (96.8)	1 (3.2)	
		Other ^1^	21 (91.3)	2 (8.7)	
		Total	69 (92.0)	6 (8.0)	

^1^ Included respondents whose stated reasons did not align clearly with either of the previous categories (indeterminate owners).

**Table 3:** Cross-tabulation of owners based on their answer to Question 1 (reason for choosing the breed) and their responses to the questions regarding awareness of health issues (Q6–Q13). The percentages are shown in parentheses.

	Questions	Groups	Yes	No	*p*
6	Are you aware that this breed may have nasal stenosis (narrow nostrils that restrict airflow)?	Aesthetics/Fashion	20 (95.2)	1 (4.8)	0.600
		Intelligence/Behavior	30 (96.8)	1 (3.2)	
		Other ^1^	23 (100)	0 (0.0)	
		Total	73 (97.3)	2 (2.7)	
7	Are you aware that these breeds may have an elongated soft palate, which can cause breathing difficulties?	Aesthetics/Fashion	18 (85.7)	3 (14.3)	0.115
		Intelligence/Behavior	31 (100)	0 (0.0)	
		Other ^1^	21 (91.3)	2 (8.7)	
		Total	70 (93.3)	5 (6.7)	
8	Are you aware that these breeds may suffer from tracheal hypoplasia (a smaller trachea), leading to breathing difficulties?	Aesthetics/Fashion	15 (71.4)	6 (28.6)	0.718
		Intelligence/Behavior	25 (80.6)	6 (19.4)	
		Other ^1^	17 (73.9)	6 (26.1)	
		Total	57 (76.0)	18 (24.0)	
9	Have you ever had specific diagnostic tests performed to assess breathing problems (e.g., X-ray, endoscopy, CT scan)?	Aesthetics/Fashion	8 (38.1)	13 (61.9)	0.144
		Intelligence/Behavior	19 (61.3)	12 (38.7)	
		Other ^1^	15 (65.2)	8 (34.8)	
		Total	42 (56.0)	33 (44.0)	
10	Are you aware that surgery may be an option to address these breathing issues?	Aesthetics/Fashion	17 (81.0)	4 (19.0)	0.190
		Intelligence/Behavior	29 (93.5)	2 (6.5)	
		Other ^1^	22 (95.7)	1 (4.3)	
		Total	68 (90.7)	7 (9.3)	
11	If so, have you already opted for surgical procedures?	Aesthetics/Fashion	6 (28.6)	15 (71.4)	0.705
		Intelligence/Behavior	12 (38.7)	19 (61.3)	
		Other ^1^	9 (39.1)	14 (60.9)	
		Total	27 (36.0)	48 (64.0)	
12	Would prior knowledge of these respiratory symptoms have influenced your decision to choose this breed?	Aesthetics/Fashion	5 (23.8)	16 (76.2)	<0.001
		Intelligence/Behavior	1 (3.2)	30 (96.8)	
		Other ^1^	11 (47.8)	12 (52.2)	
		Total	17 (22.7)	58 (77.3)	
13	Would knowledge of the costs associated with these procedures have influenced your purchasing decision?	Aesthetics/Fashion	5 (23.8)	16 (76.2)	0.080
		Intelligence/Behavior	1 (3.2)	30 (96.8)	
		Other ^1^	4 (17.4)	19 (82.6)	
		Total	10 (13.3)	65 (86.7)	

^1^ Included respondents whose stated reasons did not align clearly with either of the previous categories (indeterminate owners).

## Data Availability

The raw data supporting the conclusions of this article will be made available by the authors on request.

## Supplementary Materials

The following supporting information can be downloaded at https://www.mdpi.com/article/10.3390/ani15152288/s1: File S1: Information pursuant to article 13 of regulation (EU) 2016/679; Figure S1: Demographic chart that illustrates the distribution of dog owners, categorized by selected brachycephalic breeds and segmented by the gender of the owners.

## Abbreviations

The following abbreviations are used in this manuscript:

BOAS	Brachycephalic Obstructive Airway Syndrome
SDB	Social Desirability Bias
