# Supplementary material for: Owner Awareness, Motivation and Ethical Considerations in the Choice of Brachycephalic Breeds: Evidence from an Italian Veterinary Teaching Hospital Survey
Source: Animals (Basel). 2025 Aug 5;15(15):2288. doi: 10.3390/ani15152288 (PMC12345429; doi:10.3390/ani15152288)

Figure S1. Demographic chart that illustrates the distribution of dog owners, categorized by selected brachycephalic breeds and segmented by the gender of the owners.

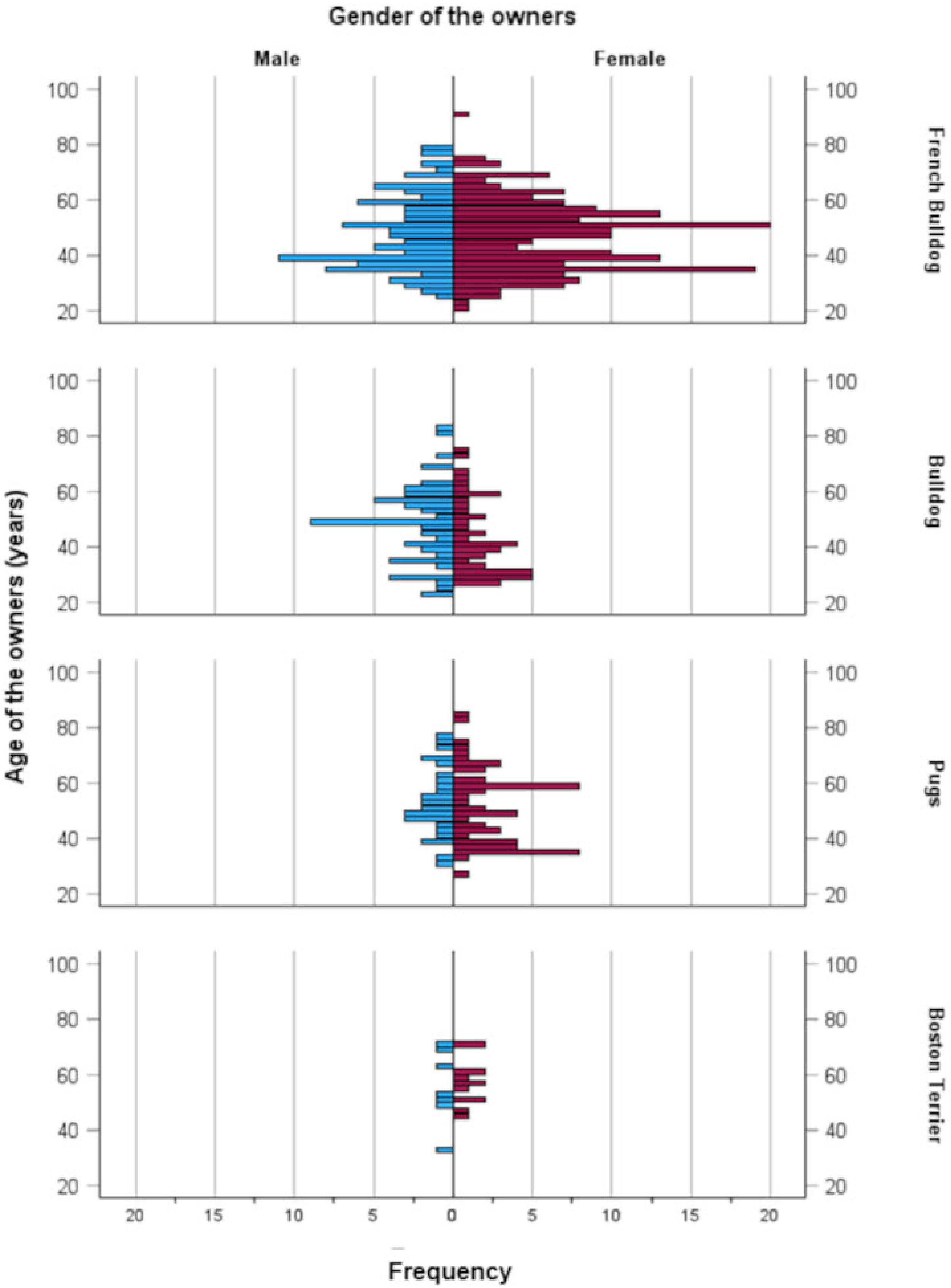

Supplement: Supplementary file 1 [file animals-15-02288-s001.zip › animals-3757806-supplementary.pdf]
