# Supplementary material for: Owner Awareness, Motivation and Ethical Considerations in the Choice of Brachycephalic Breeds: Evidence from an Italian Veterinary Teaching Hospital Survey
Source: Animals (Basel). 2025 Aug 5;15(15):2288. doi: 10.3390/ani15152288 (PMC12345429; doi:10.3390/ani15152288)
Supplement: Supplementary file 1 [file animals-15-02288-s001.zip › File S1.pdf]

INFORMATION PURSUANT TO ARTICLE 13 OF REGULATION (EU) 2016/679

This document fulfills an obligation set out by Regulation (EU) 2016/679, which, under Article 13, requires that data subjects be provided with information regarding the processing of their personal data. The legal basis for the processing is found in Article 6, letter "e" of Regulation (EU) 2016/679.

Data Controllers and Parties Involved in Processing

The personal data provided will be processed by:

- **Data Controller:** *Alma Mater Studiorum – University of Bologna* (hereinafter "Controller"), with registered office at Via Zamboni, 33 – 40126 Bologna, represented by the Rector as legal representative.
  Contact details:
  Email: privacy@unibo.it
  Certified email (PEC): scriviunibo@pec.unibo.it
- **Internal Data Processor** responsible for responding to data subjects: *the Medical Director of the "G. Gentile" University Veterinary Hospital* (hereinafter "Processor").
  Contact details:
  Medical Directorate, Department of Veterinary Medical Sciences – Via Tolara di Sopra 50, 40064 Ozzano dell'Emilia (BO)
  Fax: 051/2086178
  Email: direzionesanitaria.vet@unibo.it
- **Authorized Personnel**: staff affiliated with the Department of Veterinary Medical Sciences, authorized to process personal data in various capacities.

Contact details of the Data Protection Officer (DPO/RPD):
Email: privacy@unibo.it
Certified email (PEC): scriviunibo@pec.unibo.it

Purpose and Methods of Processing

Given that the Department of Veterinary Medical Sciences carries out clinical activities for educational and scientific purposes, all personal data of animal owners will be processed strictly for the performance of institutional objectives, in compliance with principles of fairness, lawfulness, transparency, and protection of confidentiality and data subject rights.

1. **The data provided by the data subject**, including any information attributable to them concerning the animals receiving clinical services, will be collected for purposes strictly related to clinical healthcare activities and thus processed for:
   a) Billing of healthcare services
   b) Legal reporting to Veterinary Police authorities
2. **The data provided**, including any information attributable to the data subject concerning the animals, will also be processed for purposes strictly related to the institution's educational activities, and thus for:
   a) Presentation of clinical cases to students by the Veterinary Medical staff
   b) Legal reporting to Veterinary Police authorities
3. **The data provided**, including any related information about the clinical cases, will also be processed for the institution's scientific research purposes.

4. **Biological samples** collected for clinical/diagnostic purposes may be used for educational and scientific research purposes within institutional activities.
5. **With prior consent**, the data may be stored and processed to contact the data subject—either by phone or email—for the purpose of gathering additional data and information related to research on their animals (e.g., post-discharge clinical progression, living environment, diet, etc.).

The user is also informed that, due to the educational purpose of the clinical activities carried out at our facility, practical procedures on animals aimed at diagnosing and managing medical conditions may be performed by students, under the direct supervision and expertise of medical staff.

The processing of the data will be carried out through paper and/or electronic storage. The clinical data subject to processing will be stored and controlled, in accordance with the knowledge acquired based on technical progress, the nature of the data, and the specific characteristics of the processing. The aim is to minimize, through the adoption of appropriate and preventive security measures, the risks of destruction or accidental loss of the data, unauthorized access, or processing that is not allowed or inconsistent with the purposes of collection.

Data Retention Period

The data will be retained by Alma Mater Studiorum – University of Bologna, in accordance with the principles outlined in Article 5 of Regulation (EU) 2016/679, for a period not exceeding the achievement of the purposes, with specific reference to the storage limitation principle under Article 5, letter (e).
Given the nature of the activities carried out in the context of Public Administration, the retention of data is unlimited.

Nature of Data Provision

Providing data is mandatory for the purposes of performing clinical, educational, and scientific activities. Therefore, unless in cases of urgency, refusal to provide such data will result in the inability to deliver the requested clinical services.

Rights of the Data Subject

The individual to whom the personal data refers has the rights outlined in Sections 2, 3, and 4 of Chapter III of Regulation (EU) 2016/679.

In particular, the data subject has the right to request from the Data Controller:

- Access to their personal data
- Rectification of inaccurate data
- Erasure of the data
- Limitation of processing
- Objection to processing
- Data portability

Additionally, the data subject has the right to lodge a complaint with a supervisory authority.

These rights can be exercised by writing to the Data Processing Officer at:

Medical Directorate – Department of Veterinary Medical Sciences
Via Tolara di Sopra No. 50 – 40064 Ozzano dell'Emilia (BO)
Fax: 051/20986178
Email: direzionesanitaria.vet@unibo.it
